# A Highly Integrated C-Band Feedback Resistor Transceiver Front-End Based on Inductive Resonance and Bandwidth Expansion Techniques

**DOI:** 10.3390/mi15020169

**Published:** 2024-01-23

**Authors:** Boyang Shan, Haipeng Fu, Jian Wang

**Affiliations:** 1 School of Microelectronics, Tianjin University, Tianjin 300072, China; 2021232052@tju.edu.cn; 2 Qingdao Institute for Ocean Technology, Tianjin University, Qingdao 266200, China; 3 Shandong Engineering Technology Research Center of Ocean Information Awareness and Transmission, Qingdao 266200, China

**Keywords:** GaAs, SPDT, LNA, C-band, UWB

## Abstract

This paper presents a highly integrated C-band RF transceiver front-end design consisting of two Single Pole Double Throw (SPDT) transmit/receive (T/R) switches, a Low Noise Amplifier (LNA), and a Power Amplifier (PA) for Ultra-Wideband (UWB) positioning system applications. When fabricated using a 0.25 μm GaAs pseudomorphic high electron mobility transistor (pHEMT) process, the switch is optimized for system isolation and stability using inductive resonance techniques. The transceiver front-end achieves overall bandwidth expansion as well as the flat noise in receive mode using the bandwidth expansion technique. The results show that the front-end modules (FEM) have a typical gain of 22 dB in transmit mode, 18 dB in receive mode, and 2 dB noise in the 4.5–8 GHz band, with a chip area of 1.56 × 1.46 mm^2^. Based on the available literature, it is known that the proposed circuit is the most highly integrated C-band RF transceiver front-end design for UWB applications in the same process.

## 1. Introduction

Since the Federal Communications Commission (FCC) issued an agreement on the definition of UWB, it has been a hotspot for research and development in the field of wireless communications, in which CH5 (6.24–6.7392 GHz) and CH2 (3.774–4.2432 GHz) are widely used in intelligent navigation, indoor positioning, deep-well operations supply chain management, etc. [1,2,3,4,5,6,7,8,9,10]. Compared with wireless communication technologies, such as Bluetooth and Wi-Fi, UWB systems have unique advantages in terms of positioning accuracy, communication distance, anti-interference, and high efficiency. Among them, the UWB positioning system mainly realizes high-precision positioning using the Time Difference Of Arrival (TDOA) method, in which the positioning tag sends nanosecond pulse signals to the surrounding area and calculates the positioning information via the arrival time of the pulse signals. The bandwidth of the RF transceiver front-end determines the range of the transmitted and received pulse signals of the UWB system, thus affecting the positioning accuracy of the system. In addition, with the development of technology, UWB systems have higher requirements for positioning accuracy and communication distance; the receiving path needs to have a higher sensitivity and dynamic range, and the transmitting path needs to have a higher transmitting power. In order to meet the different needs of the transmitting mode and receiving mode, the design methods of these two modes tend to be quite different, and even use different processes for the design, and the addition of auxiliary circuits such as control circuits and bias circuits makes the design of the transceiver front-end more complex, which greatly increases the difficulty of transceiver front-end integration. Therefore, the design of the transceiver front-end needs to make a reasonable trade-off from the three aspects of performance, integration area, and cost, and how to design a low-cost, highly integrated broadband design has become an urgent problem for the current UWB positioning system.

There are two basic solutions; the first is the CMOS-based highly integrated and low-cost transceiver front-end reported in [11,12,13,14,15,16,17,18]. Thanks to the small size of CMOS and complementary types of transistors, CMOS-based LNA designs for the receiver branch can achieve miniaturized broadband designs with stable bias and control circuits. In [12], a positive feedback input matching network is used to reduce the noise while realizing the bandwidth expansion, but its noise figure is largely limited by the transconductance of the transistors, which results in a suboptimal noise figure. In contrast, refs. [13,14,15,16,17,18] use different topologies, such as those cascaded and differential, to expand the bandwidth of the LNA. However, even if CMOS has more optional topologies under the same integration area, the noise, linearity, and gain are not ideal due to the loss and carrier mobility of the silicon substrate and are often only applicable to the design of the receive link, which does not satisfy the high-power requirements of the transmitter branch. The other solution is the multi-module packaging system based on the III-V high electron mobility semiconductors reported in [19,20,21,22], but due to its inherent instability and the lack of an ideal active device similar to the PMOS complementary type, this results in its low integration and large chip size. Compared to CMOS, the high electron mobility of III-V semiconductor materials compensates for the poor performance of CMOS, though there are power consumption limitations and trade-offs between gain fluctuations and bandwidth [23]. However, in conclusion, III-V materials produce a better performance and are better able to meet the high-power requirements of the transmitter branch. Among them, the GaAs pHEMT process is more mature and less costly than the GaN and GaAs heterojunction bipolar transistor (HBT) process, and finally, the GaAs 0.25 μm pHEMT process is selected as the material for this design.

In addition, the isolation within the FEM is essential. The basic approach is to realize the switching and isolation of the transmitting mode and receiving mode using the SPDT structure described in [22]. However, conventional SPDT switches often need to sacrifice their insertion loss performance if they want to achieve high isolation under defined bias conditions.

**Figure 1:**
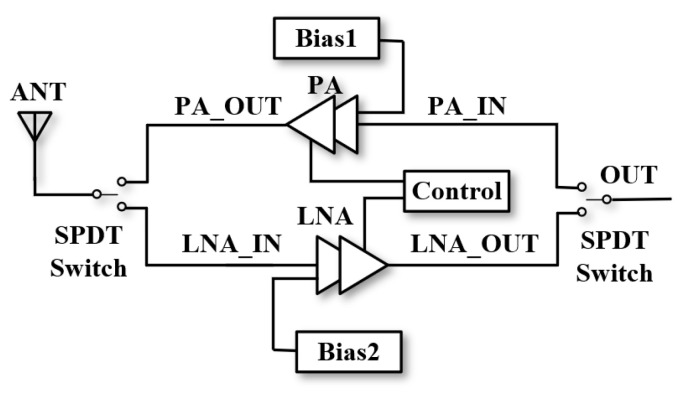
RF transceiver front-end topology.

In this paper, a low-cost, highly integrated RF transceiver front-end design is proposed based on the GaAs 0.25 μm pHEMT process with the topology shown in Figure 1, which incorporates two SPDTs, an LNA and a PA with their active bias and matching components. There are two common terminals, ANT and OUT, corresponding to the input of the LNA and the input of the PA. The T/R mode is switched using two control pins with voltages of +3.3 V/0. The switch is improved on the traditional SPDT structure by adding a resonant inductor, which improves the isolation capability of the SPDT and reduces the crosstalk between the transmitting mode and receiving mode through the high-resistance resonance generated by the resonant inductor and the parasitic total capacitance of the corresponding branch, thus ensuring the stability of the system, while the transceiver front-end expands the low-frequency bandwidth through bandwidth expansion techniques using capacitive compensation, peaked inductance to compensate for the high-frequency gain, negative feedback loops and inter-stage matching to achieve in-band gain flatness, thus ultimately realizing overall bandwidth expansion as well as flat noise in the receiving mode, and reasonably allocating power consumption to ensure high linearity. Additionally, this design uses a relatively negative voltage method to control the switch switching, avoiding the introduction of additional CMOS control circuits. Its own bias circuit can provide stable active bias, avoiding the cost of an external power control chip and improving the overall integration of the transceiver front-end. The result shows that in the 4.5–8 GHz receiving mode, the typical gain is 18 dB, the noise is 2 dB, and the typical PA gain is 22 dB. Compared with the same type of front-end module, it has higher integration and gain.

## 2. Switch Design

**Figure 2:**
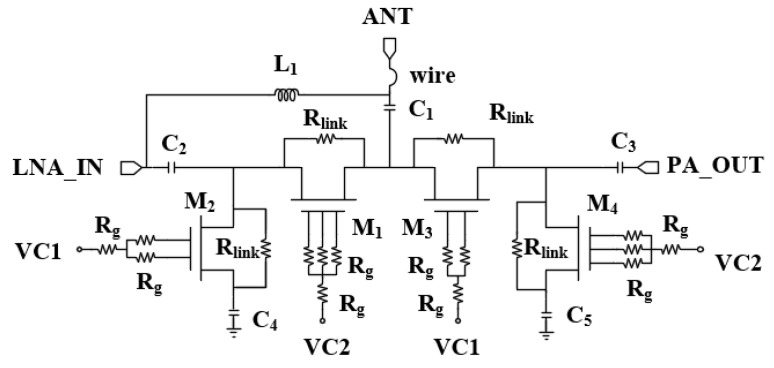
ANT port SPDT circuit schematic.

Since the two SPDTs have the same architecture, the switch at the ANT port is introduced as an example in this paper. The switching topology is shown in Figure 2, where the signal is transmitted by wire bonding to the ANT common port, and the other two ports of the SPDT are connected to the input of the LNA (LNA_IN) and the output port of the PA (PA_OUT). Capacitors for DC isolation and matching are provided in front of each port, and the ground port and parallel Inductive Resonance Matching L_1_ is incorporated to optimize the switching isolation to reduce inter-module effects. There are a total of four depletion transistors; the source-drain terminals of each transistor are connected through the source-drain resistor R_link_, and the gate is connected to the corresponding control bits VC1 and VC2 through the resistor R_g_. In the receiving mode, the transistors M_1_ and M_4_ are turned on, M_2_ and M_3_ are in the cutoff region, while in the transmitting mode, the transistors are in opposite states.

The switch switches modes via relative negative voltage technology. The gate-source voltage VGS of the depletion transistor is −0.8 V. The gate of the transistor in the on state is connected to a positive potential of 3.3 V, and due to the parasitic resistance of the transistor itself and the loss of R_g_, the voltage of the common node becomes 2.96 V, the gate potential of the transistor in the cutoff area is 0 V, and its corresponding VGS is −2.96 V, which realizes the relative negative voltage shutdown to avoid the use of negative voltage charge pumps and saves the cost of external CMOS control circuits. The SPDT utilizes an asymmetric structure to achieve the different requirements of insertion loss and isolation for the T/R mode. At the same time, the multiple-gate structure of each transistor reduces the interconnections between the drain and source, minimizing transistor distortion and providing a sufficient linearity output for the T/R mode.

**Figure 3:**
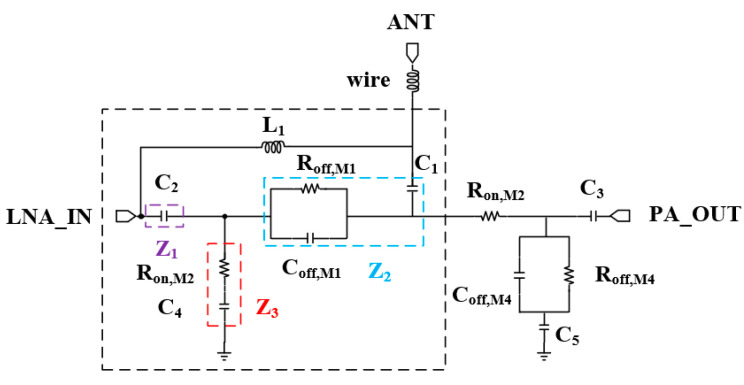
ANT port SPDT equivalent circuit diagram in the transmitting mode.

Fully integrated circuits have high requirements for switch isolation. Poor switching isolation can cause the input and output port impedance of the LNA and PA to deviate from 50 Ω, especially in the transmitting mode, and severe cases can affect the stability of the PA, leading to system self-excitation [24,25]. However, improved isolation leads to the deterioration of insertion loss. In this paper, L_1_ is introduced on the basis of the conventional SPDT structure to improve the isolation degree in the transmitting state via the Inductive Resonance Matching technique. According to the equivalent model of the switch in on and off modes introduced in [26], the isolation degree in the transmitting mode is related to the magnitude of C_off,M1_ and R_off,M1_ of the transistor in the M1 cutoff state and R_on,M2_ of the transistor in the M_2_ on state, who determine the degree of signal leakage, and the equivalent model is shown in Figure 3.

**Figure 4:**
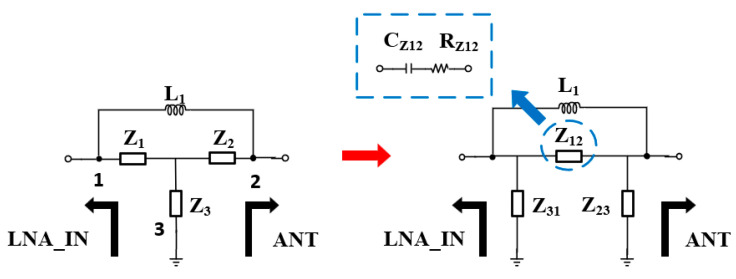
Y-network impedance conversion.

By converting the M_1_ shutdown equivalent circuit and capacitor C_1_ to a series equivalent capacitor C^’^_1_ and series equivalent resistor R^’^_off_ circuit through series–parallel conversion, the impedance Z_1_ is obtained, C_2_ to form the impedance Z_2_, R_on,M2_ and the impedance of the capacitor C_4_ is denoted as Z_3_. Converting the Y-type network into the network shown in Figure 4 yields the impedance of Z_12_ as Equation (1).

(1) $$\begin{array}{cc} Z_{12} & =Z_{1}+Z_{2}+\frac{Z_{1}Z_{2}}{Z_{3}}\\ & =R_{off}^{\prime }+\frac{1}{\mathrm{jw}C_{1}^{\prime }}+\frac{1}{\mathrm{jw}C_{2}}+\frac{\left(R_{off}^{\prime }+\frac{1}{\mathrm{jw}C_{1}^{\prime }}\right)\frac{1}{\mathrm{jw}C_{2}}}{R_{on,M2}+\frac{1}{\mathrm{jw}C_{4}}}\\ & \approx R_{off}^{\prime }+\frac{C_{4}}{C_{1}^{\prime }}R_{off}^{\prime }-\frac{C_{4}^{2}}{C_{1}^{\prime }C_{2}}R_{on,M2}+\frac{1}{\mathrm{jw}C_{1}^{\prime }}+\frac{1}{\mathrm{jw}C_{2}}+\frac{C_{4}}{\mathrm{jw}C_{1}^{\prime }C_{2}}\\ & =R_{Z_{12}}+\frac{1}{\mathrm{jw}C_{Z_{12}}} \end{array}$$

For ease of calculation, orders of magnitude too small for Z_12_ are rounded off, and the series equivalent capacitance C_Z21_ and the equivalent resistance series R_Z12_ are obtained; Z_12_ forms a parallel resonance with the inductor L_1_, and the frequency of the LC resonance high-resistance state resonance is given as Equation (2).

(2) $$f=\frac{1}{2\pi }\sqrt{\frac{C_{Z_{12}}}{L_{1}\left(C_{Z_{12}}^{2}+R_{Z_{12}}^{2}\right)}}$$

**Figure 5:**
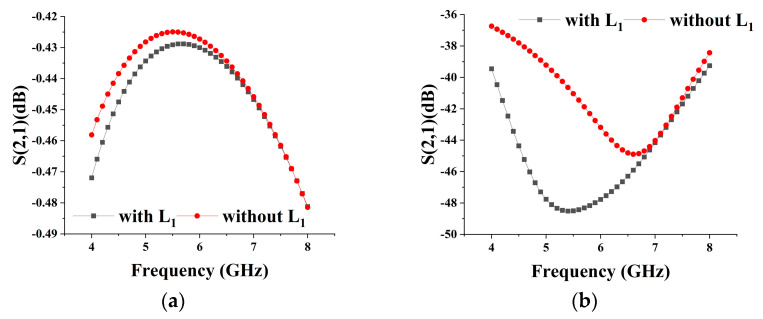
(**a**) Insertion loss of SPDT with and without L_1_ in receiving mode; (**b**) Isolation of SPDT with and without L_1_ in transmitting mode.

Since the value is limited by the requirements of the standing wave characteristics of the LNA in the receiving mode, the value of L_1_ at a fixed frequency is mainly affected by the size of the M_1_ transistor as well as the influence of Z_3_. Meanwhile, the insertion loss in the receiving mode is also considered as the basis for the selection of the size, and finally, M_1_ is selected as 9 × 100 μm, M_3_ as 5 × 75 μm, and C_4_ as 2.8 pF. Figure 5 shows the comparison of the isolation in the transmitting mode and the insertion loss in the receiving mode with and without L_1_, respectively. It can be seen that the addition of the resonant inductor optimizes the isolation degree, and the effect on the insertion loss is more minor at low frequencies and has no effect on the high-frequency part. The inclusion of L_1_ can increase the isolation between the transmitting and receiving modes, thus improving the stability of the overall system and ensuring that normal operation in any one mode is not affected by other modes.

## 3. LNA Design

**Figure 6:**
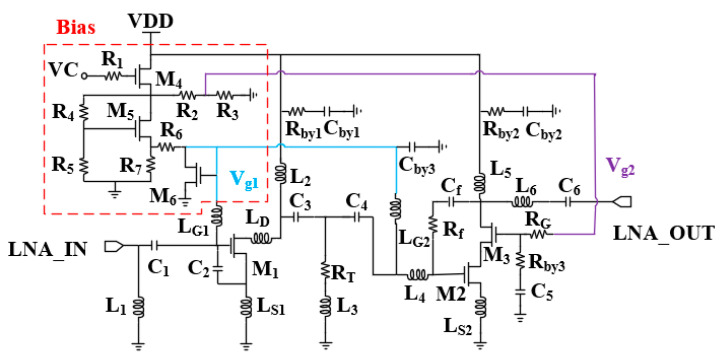
Circuit topology of LNA.

The LNA circuit consists of two stages in cascade, and the schematic is shown in Figure 6. The first stage adopts a common-source topology with a gate-source compensating capacitor C_2_ and a source-degradation inductor L_S1_ to extend the low-frequency bandwidth and obtain good noise performance. The input matching is high-pass-filtered, and the output matching uses a peaked inductor to compensate for the high-frequency gain. The interstage matching uses a T-shaped matching network to realize inter-stage broadband matching. The second stage adopts a common source and common gate structure, and a series–parallel peaked inductor is added to the output to compensate for the gain roll-off at high frequencies. In addition, a negative feedback network with the feedback resistor R_f_ and feedback capacitor C_f_ are added to optimize the flatness while improving the stability of the overall LNA circuit. The final stage of the amplifier circuit has the most significant impact on the linearity of the overall circuit. The second stage, by selecting a larger enhancement transistor size, allows the LNA to have a higher gain in the high-frequency section with a better linearity output. The gate voltage of the LNA is provided by the active biasing of the transistor, and M_4_ controls the operating state of the circuit. The active bias prevents the deterioration of linearity at high power. In order to ensure sufficient stability, the circuit suppresses oscillations by adding a resistor in front of the bypass capacitor to improve stability.

In order to ensure that the LNA has flat gain and noise at 4.5–8 GHz, the LNA is broadband-expanded in three ways, namely, the compensation capacitor for the input matching of the first stage and expanding the low-frequency bandwidth, the series–parallel-peaked inductor for compensating the high-frequency gain, and input matching and interstage matching in the form of a negative-feedback network and a high-pass for adjusting the flatness. Each of these three methods is described below.

**Figure 7:**
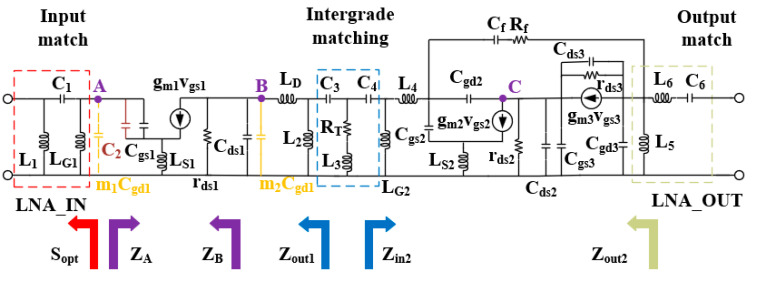
Small-signal circuit modeling for LNA.

The small signal model of the LNA circuit is shown in Figure 7. Due to power consumption limitations, in order to achieve good noise and gain for the LNA circuit, the first stage transistor size is considered to be a small-size low-power design, which is mainly optimized for low-frequency gain and noise, and the second stage is performed primarily to compensate for the linearity and high-frequency gain for large power consumption. The bandwidth of common-source amplifiers is limited due to the parasitic capacitance C_gs1_ and the effect of m_1_C_gd1_ from the Miller effect. Since the value of m1Cgd1 is too small, it is ignored for ease of calculation. Assuming a gate parasitic resistance of R_g_, the quality factor Q of the transistor’s input impedance can be expressed as (wC_gs1_R_g_)^−1^. The small size of the transistor results in a very small C_gs1_, which limits the bandwidth of the signal in the low-frequency section. An enhancement field effect transistor with a core of 2 × 50 μm is selected to compensate for the decrease in C_gs1_ in the low-frequency band by adding a compensation capacitor C_2_. 

**Figure 8:**
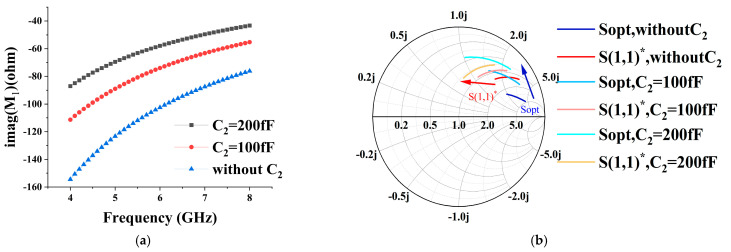
(**a**) Imaginary part impedance of the transistor for different values of C_2_; (**b**) Smith’s circle plot of S(1,1)* and Sopt variations for different values of C_2_.

Corresponding to the different compensation capacitance transistor input impedance, an imaginary part of the change is shown in Figure 8a, and it can be clearly seen that as the value of C_2_ becomes larger, the transistor’s low-frequency band impedance change is gentler, and the Q value decreases, so as to realize the bandwidth of the low-frequency band which expands.

While analyzing from the point of view of noise, both the real and imaginary parts of optimal noise matching are inversely proportional to C_gs1_, and the addition of the compensation capacitor also makes the optimal noise impedance decrease but does not deteriorate the minimum noise figure [27]. According to the noise cascade equation in [28], it can be seen that the noise of the first stage has the most significant impact on the LNA. To keep the overall noise at a small and flat value, making the minimum noise of the impedance electrode coincide with the maximum gain impedance point is the ideal situation. However, the difference between these two points is usually large. At this point, the addition of the source-level degradation inductor L_S1_ is very necessary, which not only reduces the gap between the two impedance points but also improves the stability of the circuit. Figure 8b shows the Smith chart of the 4–8 GHz input port conjugate S(1,1)* and the optimum source reflection coefficient Sopt for different values of C_2_ under the value of the degenerate inductor of 300 pH. Through the results, it can be found that the compensation capacitance narrows the high and low-frequency gap between the input impedance and the optimal noise source impedance under the effect of L_S1_, and the two curves’ impedances gradually converge to 50 Ω with an increase in the compensation capacitance, which is finally selected to be 200 fF in size.

The second method is the series and parallel inductance peaking technique; the output of the first stage of the common source pole, due to the Miller effect, leads to a more pronounced decline in high-frequency gain. By adding peaked inductors L_D_ and L_2_ at the output, the output impedance and gain of the first stage can be expressed as Equations (4) and (5). With the increase in the frequency, the two inductors compensate for the output impedance, thus expanding the high-frequency bandwidth. The second stage is no longer added before point C for inductor compensation due to the characteristics of its cascade structure to suppress the Miller effect, and the high-frequency bandwidth is compensated by matching the access to series and parallel inductors at the output.

(3) $$Z_{B}=\left[r_{ds1}//\frac{1}{s(C_{ds1}+m_{2}C_{gd1})}\right]+\left[1+g_{m1}\left((r_{ds1}//\frac{1}{sC_{ds1}}\right)\right]\left[sL_{s1}//\frac{1}{s(C_{gs1}+C_{2})}\right]$$

(4) $$Z_{out1}=(Z_{B}+sL_{D})//sL_{2}$$

(5) $$A_{v}\approx -\frac{g_{m1}}{1+g_{m1}L_{s1}}\;Z_{out1}$$

**Figure 9:**
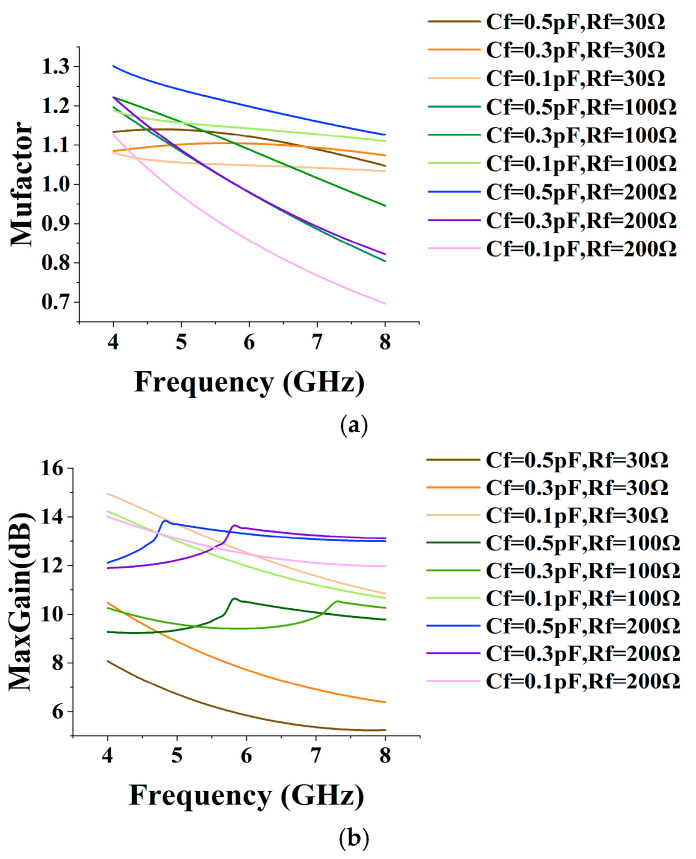
(**a**) Stability curves at different C_f_ and R_f_ (**b**) Maximum gain curves at different C_f_ and R_f_.

After the high-frequency and low-frequency bandwidths are expanded, the LNA maintains overall gain flatness by changing the matching form and adding a negative feedback network to the second stage. The capacitor C_f_ and resistor R_f_ at the common gate output and common source input of the second stage can form a feedback network that not only optimizes flatness but also increases the stability of the LNA. The maximum gain and stabilization factor are given in Figure 9. Adding the feedback capacitor, C_f_ realizes the DC isolation, and as the value of C_f_ is taken to be larger, the gain decreases as the degree of feedback increases, and the peak of the maximum gain moves towards the low frequency. Adding resistor R_f_ can cut the high-frequency gain roll-off speed; the smaller the gain roll-off in the high-frequency part, the lower the gain in the low-frequency part and the better the gain flatness. However, large resistor values can hinder high-frequency negative feedback, resulting in a lack of stability. The final choice of capacitance takes the value of 0.1 pF, and the resistance takes the value of 30 Ω. In addition, input matching and interstage matching use high-pass matching to appropriately reduce the low-frequency gain and solve the problem of gain flatness deterioration. Due to the limitation of noise matching, interstage matching with the resistor introduced is more accessible to achieve flatness optimization than input matching. It consists of C_3_, C_4_, and a resistor R_T_ in series with inductor L_3_ to form a parallel branch, which, by adjusting the equivalent resistor R_T_, can change the Q value of the interstage network to meet the requirements of different frequency bands on the gain.

## 4. PA Design

**Figure 10:**
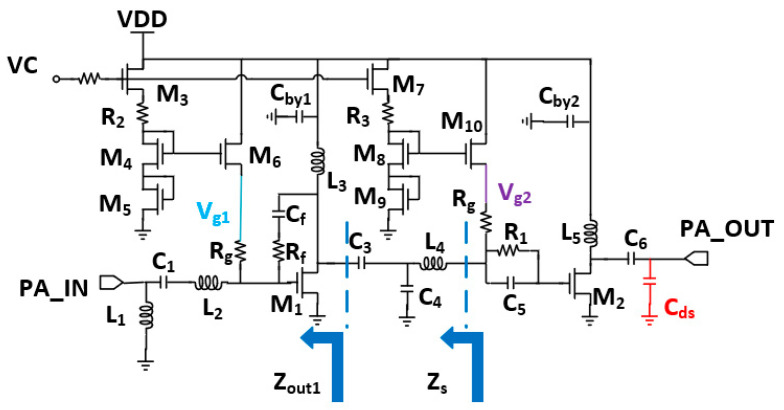
Circuit topology of PA.

The PA topology is shown in Figure 10. The PA design consists of two common source cascade stages, and the input matching consists of L_1_, L_2_, and C_1_. The addition of series resonance to impedance matching can make the high-frequency and low-frequency ends of the impedance move in opposite directions, which narrows down the impedance curve to a minimal range in order to achieve broadband input matching. The first transistor stage is used as the driver stage. To ensure the stability and flatness of the first stage, RC negative feedback is used to optimize the gain flatness as well as linearity at the expense of partial gain. The output is passed to the interstage matching network via the peaking inductor L_3_. The interstage matching consists of C_3_, C_4_, and L_4_. In order to realize the matching between the driving stage Z_out1_ of the first stage and the power stage Z_s_ of the second stage so that the output impedance curve of the first stage overlaps with the source impedance curve of the second stage, the values of several components are carefully selected to achieve high efficiency while realizing broadband matching. The second stage uses a larger transistor as the power stage to further increase the output power, and the gate is stabilized using an RC stabilization network to ensure the stability of the second stage. At the output of the circuit, since the switched receiver branch is in a high resistance state, and the parallel switch of the transmitter branch is in the cutoff state showing capacitance, the load traction is achieved using the parallel peaked inductor L_5_ and capacitor C_6,_ the parallel parasitic capacitor C_ds_, and the OP1dB which reaches 18.13 dBm, with a Power-Added Efficiency (PAE) of 31.7%. The transistors are actively biased to provide gate voltage to the circuit and provide compensation for circuit linearity, and their operating state is controlled by M_3_.

## 5. Measurement Results

**Figure 11:**
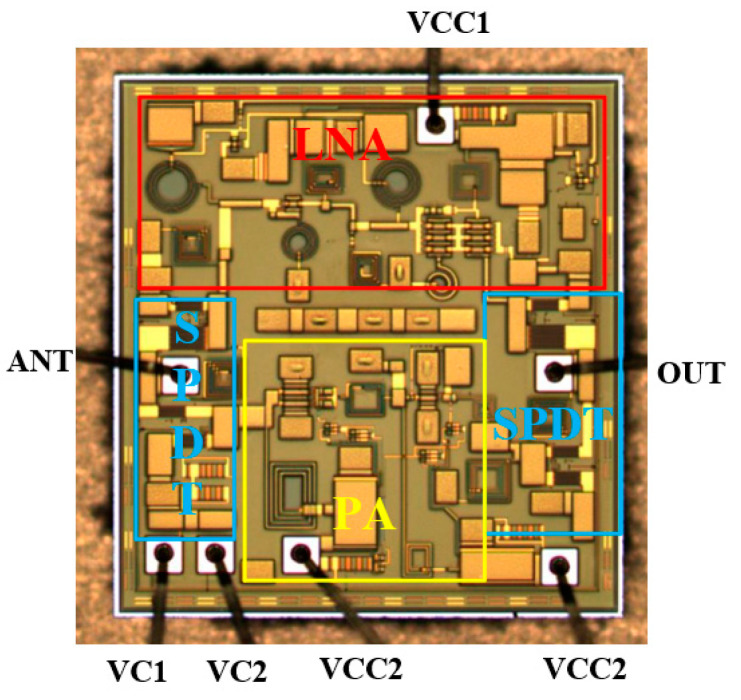
Microscope picture of the chip.

**Figure 12:**
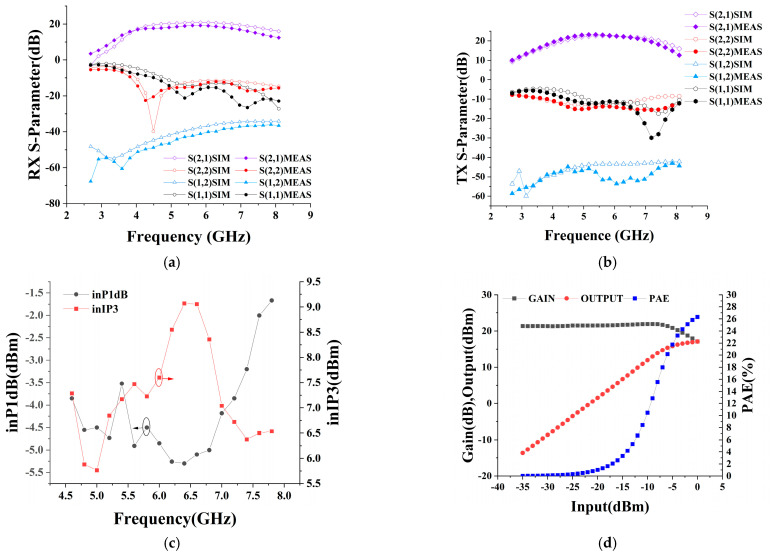
(**a**) S-parameters under RX; (**b**) S-parameters under TX; (**c**) Linearity test under RX; (**d**) Linearity vs. efficiency curve of TX at 6.2 GHz.

**Figure 13:**
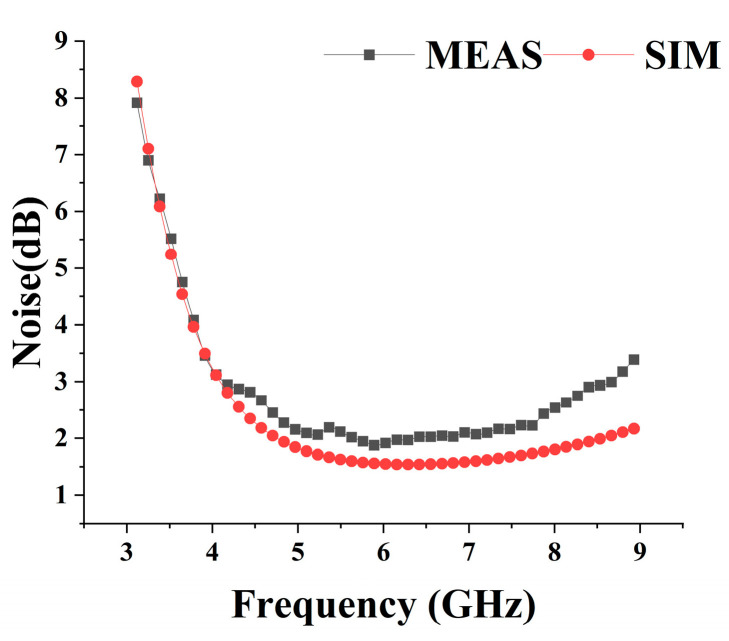
Noise curve of RX.

The microscopic picture of the chip is shown in Figure 11, and the area of the chip is 1.56 × 1.46 mm^2^. The fabricated transceiver front-end was experimentally verified by configuring it on the evaluation board. The transceiver front-end is powered by a 3.3 V power supply in both transmitting and receiving modes, and its static DC power consumption is 41.15 mA and 27.27 mA, respectively. S-parameter measurements using a network analyzer are shown in Figure 12a,b, which shows that the input/output match between receiving and transmitting modes is good in the range of 4.5–8 GHz, and the range of receiving mode gain is 14–19 dB, and that of the transmitting mode gain is 18–23 dB. In addition, the large signal test of the chip using the spectrum, including P1dB as well as IP3 results, are shown in Figure 12c; the input inP1dB of the receiving mode is higher than −5.3 dBm in the frequency band, and the input inIP3 is greater than 5.7 dBm, and the output power and PAE of the receiving mode at 6.2 GHz are depicted in Figure 12d. Relative to the input power variation, OP1dB is greater than 16.27 dBm, and the PAE is greater than 26%. The noise figure in the receiving mode is shown in Figure 13, and the receiving mode in-band noise range is 1.9–2.7 dB when tested using a noise source. The transceiver front-end has a sufficiently low noise figure and sufficient linearity. Depending on the requirements of the UWB system for different frequency bands, external appropriate filters can be selected to realize the dynamic range requirements in compliance with the receiver standards.

Table 1 summarizes previously reported RF front-ends operating at similar frequency bands and using similar processes. Compared to previously reported RF front-ends, this design integrates both RF switches, LNA, PA, the matching network, and the bias network for the highest level of integration. Compared with the traditional UWB RF front-end design, this design integrates the PA and bias circuit, and control circuit into the same FEM and does not deteriorate the performance of the receiving mode. Moreover, the chip area is almost close to the area of other RF front-ends with low integration, and the increased area and cost are much smaller than the area of the PA chip and the power management chip, so it can be considered that the design in this paper has higher integration and better switching isolation. At the same time, the transceiver front-end in the 4.5–8 GHz frequency band has low noise, high gain, a sufficient linearity guarantee, and relatively low power consumption, which can basically meet the requirements of the new generation of the UWB positioning system.

## 6. Conclusions

This paper presents a highly integrated transceiver front-end for UWB applications using the 0.25 μm GaAs pHEMT process, integrating a PA and LNA and two SPDTs, as well as matching and biasing circuits and logic control circuits. The transceiver front-end can realize both receiving and transmitting functions at the same time. The switch introduces a new inductive resonance network, which improves isolation and reduces the crosstalk between the two modes, thereby improving the stability of the whole system via generating high-resistance resonance through the resonant inductance and parasitic capacitance of the switch at the input and output. The bandwidth is expanded by the bandwidth expansion technique, which compensates for the gain at different frequency bands using compensation capacitors, peaked inductors, interstage matching, and negative feedback loops, respectively. Additionally, through the bandwidth expansion technique, the transceiver front-end achieves a typical value of 18 dB of gain in the receiving mode and −4.5 dBm of inP1dB and 7.5 dBm of inIP3, with a noise figure of 2 dB. In the transmitting mode, the typical value of gain is 22 dB, and the OP1dB can reach 16 dBm, with a power additive efficiency better than 26%. The transceiver front-end, to meet the requirements of low cost, high integration, and in both modes, can work with mutual instability as the simulation and measurement trends are basically the same. In addition, compared with other transceivers, the front-end has lower power consumption and higher gain.

However, there are still many areas of this design that need to be improved. Due to modeling and simulation errors, the actual test of the DC bias circuit of this design has a higher current than the simulation results. Together with the problem that the cascade circuit is more sensitive to the bias circuit, it leads to the second stage circuit of the receiving mode, which is used to compensate for the high-frequency gain, to be tested when shifted toward a lower frequency than the simulation. So, the issue of the robustness of the bias circuit is the key to be studied as the next step. In addition, the functions of band selection and out-of-band suppression are not purposely designed in this paper, which will also form the research direction of the next design.

## Figures and Tables

**Table 1:** Performance comparison.

References	[29]	[30]	[31]	[32]	This Work
Frequency (GHz)	0.5–4	3.5–7	1.5–2.7	3.1–8	4.5–8
Topology	LNA	LNA	LNA + SW	LNA	SW + LNA + PA
RX/TX Gain (dB)	20.5	17	17.5	4	18/23
NF (dB) for RX	4	1.8	0.75	5	2
RX/TX inP1dB (dBm)	−0.5	−3	−2.5	-	−5.3/−5
PDC (mW)	NA	400	300	-	81.81
Off-chip Components	YES	NO	YES	NO	NO
Chip Size (mm^2^)	1.78	1.97	1.95	2.1	2.28
GaAs Process	2 μm HBT	0.5 μm MESFET	0.25 μm pHEMT	0.15 μm pHEMT	0.25 μm pHEMT

## Data Availability

Data are contained within the article.

## References

[1] Cerro G., Ferrigno L., Laracca M., Miele G., Milano F., Pingerna V. (2022). UWB-Based Indoor Localization: How to Optimally Design the Operating Setup?. IEEE Trans. Instrum. Meas..

[2] Bocus M.J., Chetty K., Piechocki R.J. UWB and WiFi Systems as Passive Opportunistic Activity Sensing Radars. Proceedings of the 2021 IEEE Radar Conference (RadarConf21).

[3] Guo Y.-R., Lin M.-T., Wu Z.-F., Huang R.-Q., Liu J.-B. (2023). Amplitude-phase weighted loop for multichannel interference suppression in RF front-end. J. Radio Wave Sci..

[4] Liu X., Liu M., Zhao Z., Liu C., Zhang L. (2022). Analysis of the impact of RF receiver nonlinearity on diversity and multiplexing. J. Radio Wave Sci..

[5] Abdat M., Wan T.-C., Supramaniam S. Survey on Indoor Wireless Positioning Techniques: Towards Adaptive Systems. Proceedings of the 2010 International Conference on Distributed Frameworks for Multimedia Applications.

[6] Gresham I., Jenkins A., Kinayman N., Point R., Lu Y., Ito R., Street A. Si Based UWB Radar Sensors—Design, Development, and Production. Proceedings of the 2006 IET Seminar on MM-Wave Products and Technologies.

[7] Fernandes J.R., Wentzloff D. Recent Advances in IR-UWB Transceivers: An Overview. Proceedings of the 2010 IEEE International Symposium on Circuits and Systems.

[8] Shan X., Shen Z. (2019). Miniaturized UHF/UWB Tag Antenna for Indoor Positioning Systems. IEEE Antennas Wirel. Propag. Lett..

[9] Fredenburg J., Flynn M. A 90 MS/s 11 MHz Bandwidth 62 dB SNDR Noise-Shaping SAR ADC. Proceedings of the 2012 IEEE International Solid-State Circuits Conference.

[10] Xu Q. Design of Accurate Intelligent Positioning System through UWB Technology. Proceedings of the 2022 IEEE 4th International Conference on Civil Aviation Safety and Information Technology (ICCASIT).

[11] Wang H., Zhu Z. (2015). Energy-Efficient and Reference-Free Monotonic Capacitor Switching Scheme with Fewest Switches for SAR ADC. IEICE Electron. Express.

[12] Mehrjoo M.S., Yavari M. (2010). A New Input Matching Technique for Ultra Wideband LNAs. IEICE Electron. Express.

[13] Chen K.-H., Lu J.-H., Chen B.-J., Liu S.-I. (2007). An Ultra-Wide-Band 0.4–10-GHz LNA in 0.18-Μm CMOS. IEEE Trans. Circuits Syst. II Express Briefs.

[14] Blaakmeer S.C., Klumperink E.A.M., Leenaerts D.M.W., Nauta B. (2008). Wideband Balun-LNA With Simultaneous Output Balancing, Noise-Canceling and Distortion-Canceling. IEEE J. Solid-State Circuits.

[15] Lee Y.-C., Tarng J.-H. (2007). A Low-Power CMOS LNA for Ultra-Wideband Wireless Receivers. IEICE Electron. Express.

[16] Zhang H., Chen G.-C., Lai S.-M., Liu J.-H. (2008). A High-Gain Differential CMOS LNA for 3.1–10.6 GHz Ultra-Wideband Receivers. IEICE Electron. Express.

[17] Nam H., Park J., Park J.-D. (2018). A 1–13 GHz CMOS Low-Noise Amplifier Using Compact Transformer-Based Inter-Stage Networks. IEICE Electron. Express.

[18] Zhang K., Li W., Li N., Ren J. (2013). A 0.13-&micro;m CMOS 0.1–12 GHz Active Balun-LNA for Multi-Standard Applications. IEICE Electron. Express.

[19] Lim W.J., Kumar N., Yarman S., Chacko P. (2017). Ultra-Wideband GaN HEMT Power Amplifier with Practical Mixed Lumped Approach Employing Real-Frequency Technique. IEICE Electron. Express.

[20] Yu Y.-H., Hsu W.-H., Chen Y.-J.E. (2010). A Ka-Band Low Noise Amplifier Using Forward Combining Technique. IEEE Microw. Wirel. Compon. Lett..

[21] Zhang Y., Ma K., Yang H., Zhang Y., Guo Y. (2017). 1–20 GHz Distributed Power Amplifier Based on Shared Artificial Transmission Lines. IEICE Electron. Express.

[22] Cheng C.-S., Lin S.-W., Wei C.-C., Chiu H.-C., Yang R.-J. A High Isolation 0.15 μm Depletion-Mode pHEMT SPDT Switch Using Field-Plate Technology. Proceedings of the 2007 Asia-Pacific Microwave Conference.

[23] Wei D., Zhang J., Wu T., Ma S., Ren J. A 22–40.5 GHz UWB LNA Design in 0.15 um GaAs. Proceedings of the 2019 IEEE 13th International Conference on ASIC (ASICON).

[24] Eltaliawy A., Long J.R., Cahoon N. A DC to 43-GHz SPST Switch with Minimum 50-dB Isolation and +19.6-dBm Large-Signal Power Handling in 45-Nm SOI-CMOS. Proceedings of the 2020 IEEE Radio Frequency Integrated Circuits Symposium (RFIC).

[25] Mafinejad Y., Zarghami M., Kouzani A.Z., Mafinezhad K. (2013). Design and Simulation of a High Isolation RF MEMS Shunt Capacitive Switch for C-K Band. IEICE Electron. Express.

[26] Park J., Lee W., Hong S. (2020). A Small-Size K-Band SPDT Switch Using Alternate CMOS Structure with Resonating Inductor Matching. IEEE Microw. Wirel. Compon. Lett..

[27] Yang X., Zhang Z., Liu H., Zhang G., Wang T., Huang Y., Wang Q., Luo L. (2022). A K-Band MMIC Low Noise Amplifier in GaN-on-Si 100-Nm Technology for MIMO Radar Receivers. IEICE Electron. Express.

[28] Khosravi H., Sheikhi M., Bijari A., Kandalaft N. 3.5–9 GHz Ultra-Wideband LNA With Variable Gain and Noise Cancellation for Wireless Communication. Proceedings of the 2020 10th Annual Computing and Communication Workshop and Conference (CCWC).

[29] Syu J.-S., Wu T.-H., Meng C., Huang G.-W. Kukielka and Meyer Wideband Dual Feedback Amplifiers Using GaInP/GaAs HBT Technology. Proceedings of the 2009 Asia Pacific Microwave Conference.

[30] (2016). MAAM37000. 3.5–7 GHz Low Noise GaAs MMIC Amplifier.

[31] Yao J., Sun X., Lin B. 1.5–2.7 GHz Ultra Low Noise Bypass LNA. Proceedings of the 2014 IEEE MTT-S International Microwave Symposium (IMS2014).

[32] Wu C.-S., Chang C.-H., Lin T.-Y., Wu H.-M. A Ultrawideband 3–10 GHz Low-Noise Amplifier MMIC Using Inductive-Series Peaking Technique. Proceedings of the 2011 International Conference on Electric Information and Control Engineering.

